# Treatment of pelvic giant cell tumor by wide resection with patient-specific bone-cutting guide and reconstruction with 3D-printed personalized implant

**DOI:** 10.1186/s13018-023-04142-4

**Published:** 2023-09-01

**Authors:** Zhuangzhuang Li, Minxun Lu, Li Min, Yi Luo, Chongqi Tu

**Affiliations:** 1 Department of Orthopedics, Orthopaedic Research Institute, West China Hospital, Sichuan University, No. 37 Guoxue Road, Chengdu, 610041 Sichuan China; 2 Model Worker and Craftsman Talent Innovation Workshop of Sichuan Province, West China Hospital, Sichuan University, Chengdu, People’s Republic of China

**Keywords:** Pelvic GCT, 3D-printed implant, Patient-specific bone-cutting guide, Osseointegration

## Abstract

**Background:**

This study reports our experience in the treatment of aggressive pelvic GCT through wide resection assisted with patient-specific bone-cutting guides (PSBCGs) and subsequent reconstruction with 3D-printed personalized implants (3DPIs), aiming to present the operative technique of this method and evaluate its clinical efficacy.

**Methods:**

We retrospectively analyzed seven patients who underwent wide resection of pelvic GCT followed by reconstruction with 3DPIs from August 2019 to February 2021. There were two males and five females, with a mean age of 43 years. PSBCGs and 3DPIs were prepared using 3D-printing technology. The operational outcomes, local recurrence, radiological results, and any associated complications of this technique were assessed. And the functional outcomes were assessed according to the Musculoskeletal Tumor Society (MSTS) 93 functional score.

**Results:**

The mean follow-up time was 35.3 months (range 28–45 months). There was no intraoperative complication. Negative surgical margins were achieved in all patients. Postoperative pelvic radiographs showed that 3DPIs matched the shape and size of the bone defect. The anterior–posterior, inlet, and outlet pelvic radiograph demonstrated precise reconstruction consistent with the surgical planning. In addition, tomosynthesis‐Shimadzu metal artifact reduction technology (T-SMART) showed good osseointegration at an average of three months after surgery (range 2–4 months). There was no local recurrence or tumor metastasis. The average MSTS score was 24.4 (range 23–27) at the last follow-up. Delayed wound healing was observed in one patient, and the wounds healed after debridement. Prosthesis-related complications were not detected during the follow-up, such as aseptic loosening or structure failure.

**Conclusions:**

The treatment of aggressive pelvic GCTs through wide resection assisted with PSBCGs and subsequent reconstruction with 3DPIs is a feasible method, which provides good clinical results and reasonable functional outcomes.

## Background

Giant cell tumor (GCT) of bone is a benign bone tumor with local aggressiveness, representing approximately 5% of all primary bone tumors [1–3]. It typically occurs in the epiphyseal end of long bones [4–6], but less frequently, GCT affecting the pelvis is extremely rare, accounting for about 1.5–6% of all GCT [7, 8]. Presently, a variety of techniques have been elucidated for the management of pelvic GCT, encompassing radiation therapy [9, 10], intralesional curettage with or without adjunctive therapy [11, 12], and wide resection [13, 14]. However, the optimal treatment approach for pelvic GCT remains a subject of controversy, primarily due to the intricate anatomical characteristics of the pelvis and the variable aggressiveness exhibited by GCT.

In the early stages, intralesional curettage may serve as a surgical approach for treating pelvic GCT. This technique aims to maintain the structural integrity of the pelvis, potentially resulting in a favorable functional outcome. However, it is important to note that the incidence of local recurrence can vary significantly, ranging from 6.3 to 43% [13]. In cases where the lesion exhibits aggressive behavior and affects the adjacent soft tissue (Campanacci Grade III) [15], it is frequently advised to perform wide resection to mitigate the risk of local recurrence. Nevertheless, the primary considerations lie in the accurate excision of the lesions and the subsequent reliable reconstruction of the pelvic defect. Firstly, the surgical procedure requires removing the lesion with adequate margins and also to preserve as much bone stock as possible. In addition, reliable reconstruction of the affected bone is another key to restoring the pelvic biomechanical relationship and maintaining good function for a long time. However, traditional technologies still make it difficult to achieve these goals satisfactorily.

In recent years, three-dimensional (3D)-printing technology has been widely used in orthopedic surgery for tumor resection and bone defect reconstruction, including patient-specific bone-cutting guides (PSBCGs) [16, 17] and 3D-printed personalized implants (3DPIs) [18–20]. PSBCGs allow for the resection of the tumor safely while preserving bone stock as much as possible, while 3DPIs allow for the perfect matching of implants with bone defects, which seems an appealing treatment option for patients with aggressive pelvic GCT.

This study reports our experience in the treatment of aggressive pelvic GCT through wide resection assisted with patient-specific bone-cutting guides (PSBCGs) and subsequent reconstruction with 3D-printed personalized implants (3DPIs), aiming to present the operative technique of this method and evaluate its clinical efficacy.

## Methods

**Fig. 1:**
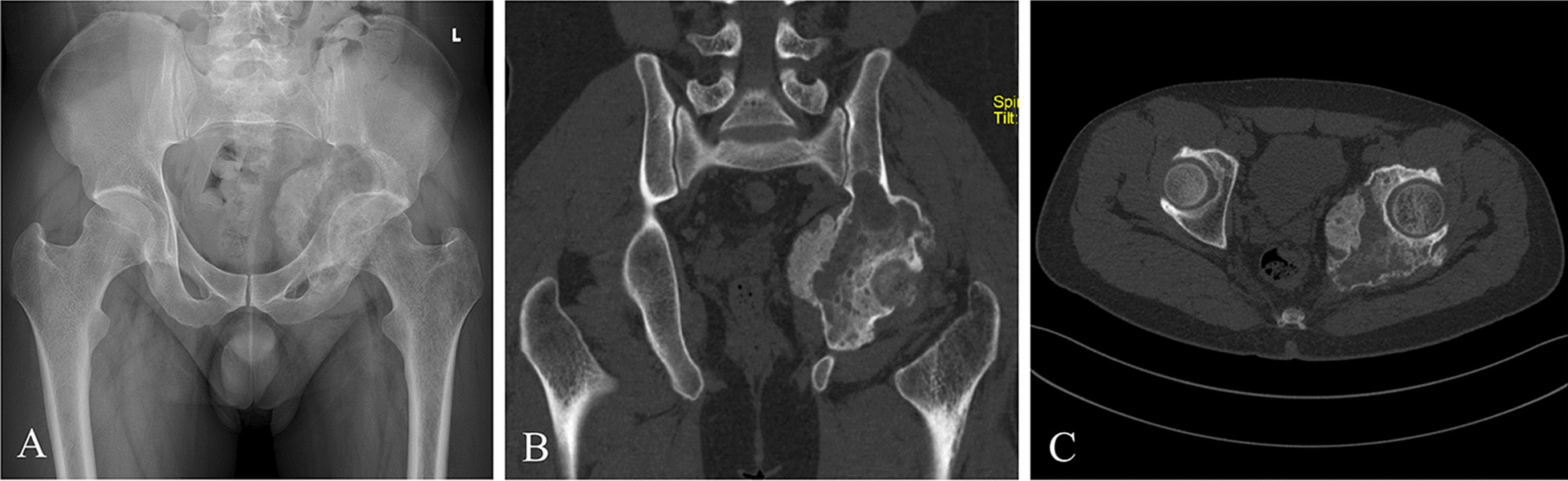
Preoperative images of a 29-year-old patient. Anteroposterior X-ray (**A**) and CT scans (**B**, **C**) showed that the lesion centered on area II Enneking, but with extension including areas I and III

Seven patients who underwent wide resection assisted with PSBCGs for pelvic GCT followed by reconstruction with 3DPIs were identified from August 2019 to February 2021. There were two males and five females, with a mean age of 43 years. All the patients meet the following criteria: (1) definite pathological diagnosis of GCT; (2) no pelvic neurovascular involvement; (3) complete clinical and radiographic data; and (4) with a minimum follow-up of 24 months after surgery. Indications for wide resections and reconstruction with 3DPIs: lesions destructed the cortical bone and affected the adjacent soft tissue (Campanacci Grade III, aggressive), with pelvic instability and discontinuity. The patients underwent plain radiography, computerized tomography (CT), and magnetic resonance imaging (MRI) of the pelvis (Fig. 1). The involved region was recorded according to the Enneking classification [21]. Preoperatively, all patients received denosumab 120 mg subcutaneously every 4 weeks (additional doses on days 8 and 15 of the first cycle). In addition, the total times of receiving denosumab before surgery are detailed in Table 1.

**Table 1:** Demographics, clinical data, and follow-up results of seven patients

Patients	Gender	Age (year)	Location^a^	Grade^b^	Total times of receiving denosumab	Follow-up, (months)	MSTS 93 score	Complications	Recurrence or metastasis
1	Female	37	I	III	4	45	25	NA	NA
2	Female	58	I	III	5	38	27	NA	NA
3	Male	23	I	III	4	35	26	NA	NA
4	Female	59	I + II	III	8	32	23	NA	NA
5	Female	43	II + III	III	6	39	23	Delayed wound healing	NA
6	Male	29	I + II + III	III	10	30	24	NA	NA
7	Female	50	II + III	III	5	28	23	NA	NA

^a^According to the classification system of Enneking and Dunham

^b^According to the Campanacci grading system

**Fig. 2:**
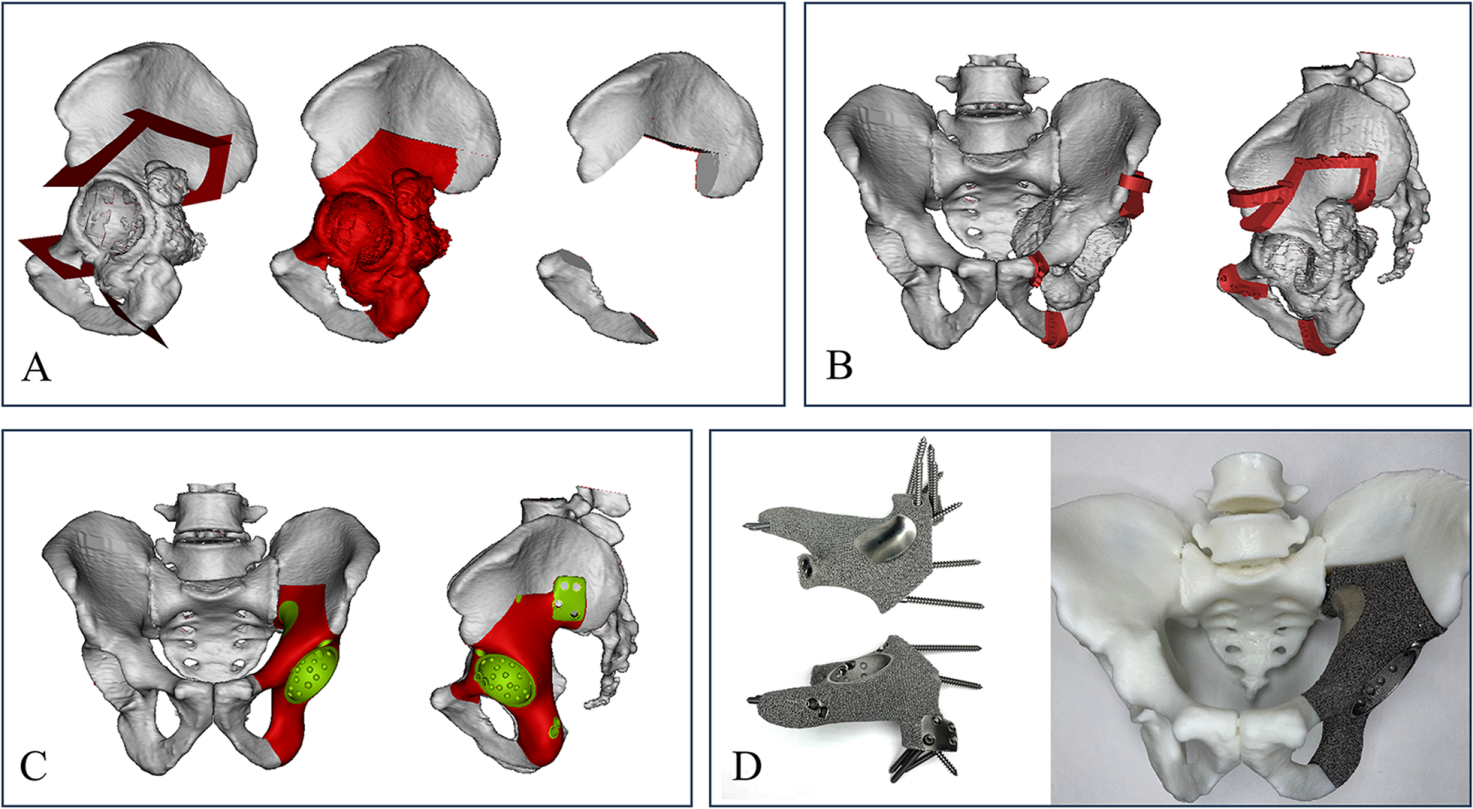
Profile of design procedure of PSBCG and 3DPI. **A** multiplanar osteotomy was planned; **B** 3D models of the PSBCG; **C** 3D models of 3DPI; **D** physical photographs of the 3DPI

The resection plains were planned with the software MIMICS (Materialise, Leuven, Belgium) (Fig. 2). Firstly, CT data was imported into this software to reconstruct a 3D model of the pelvis. The surgical margins and osteotomy plains were determined and simulated on the 3D model. “Multiplanar osteotomy with limited margins” was used to preserve bone stock as much as possible [14, 22], along with the removal of the tumor with safe margins. And PSBCP was designed consistent with the multiple osteotomy plains, and its internal surface was designed to fit the cortical bone. After that, several holes were set on the PSBCP to fix it during osteotomy. The preliminary implant was designed by mirroring the corresponding part of the opposite side. After obtaining implant model, fixation optimization was carried out, such as adding screws, and plate. The long axis of the screw aligned with the mechanical conduction and pressurized against the bone–implant interface. Wide resection assisted with PSBCG and reconstruction with 3DPI were simulated on the 3D model.

All the 3DPIs, PSBCGs, and pelvic models were manufactured by Chunli Co., Ltd. (Beijing, China). The 3DPIs were fabricated using an electron beam melting (EBM) machine (Arcam Q10), and the printing material was titanium alloy (Ti-6Al-4V). PSBCGs were manufactured from nylon powder by selective laser sintering. In addition, resin models of the pelvis with lesions and simulated resection were prepared. Preoperatively, surgeons further verified whether the PSBCGs were fitting correctly with the pelvis, and planned the procedure of reconstructing the defects with the 3DPIs. It took approximately 2 weeks to produce these devices. In detail, after obtaining patient radiological examination data, modeling and prototype of the implant were completed within 2–3 days. Afterward, discuss with senior doctors the optimization of the implant design, which took 2–3 days to complete. Therefore, the time for implant design was approximately 4–6 days. And then, production and post-treatment of the implant took about a week.

**Fig. 3:**
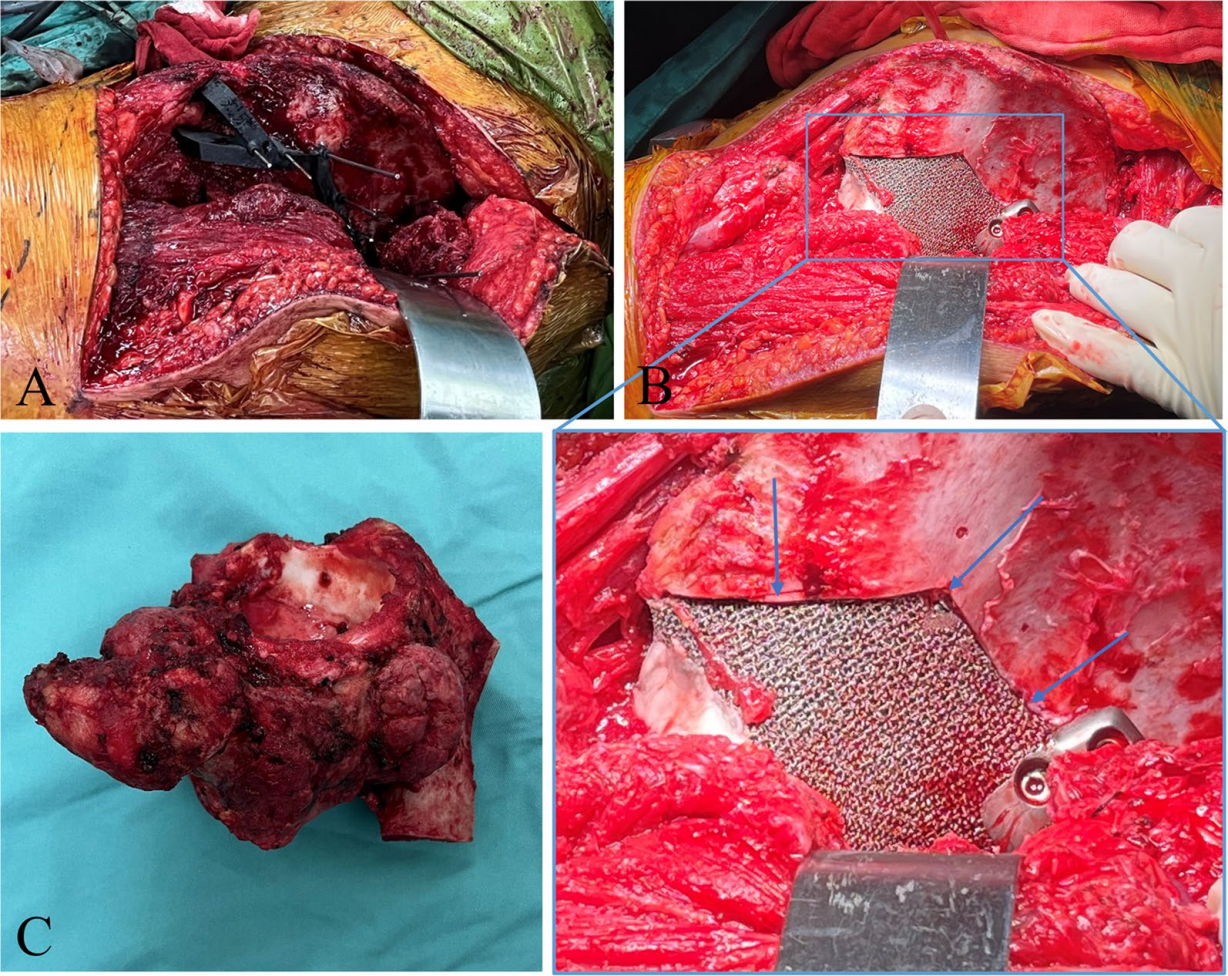
Intraoperative pictures of one case. **A** the PSBCG was installed, and K-wires were inserted into the holes of the PSBCG for fixation; **B** 3DPI was implanted consistent with the surgical planning, and matched the defect perfectly (blue arrows); **C** a photograph of the resected specimen

All the surgeries were performed by the same senior surgeon (CQ T). After sufficient exposure to the lesion, the PSBCG was installed, ensuring that the positioning module was attached to the anatomy of the pelvis (Fig. 3). K-wires were inserted into the holes of the PSBCG for fixation. The tumor was removed completely along the border of the PSBCG using a reciprocating saw. And then, the 3DPI was implanted according to preoperative planning. The specimens were washed and photographed, after which were submitted for pathological examination and resection margin status. Surgical-related outcomes such as length of operation and estimated blood loss were recorded.

Postoperatively, the patients were followed up monthly for the first three months and every three months thereafter. Pelvic radiographs (including anteroposterior, inlet, and outlet) were performed immediately after surgery and at each visit. The patients underwent CT and MRI of the pelvis every three months to detect the local recurrence. Chest CT was performed every three months to detect metastasis. In addition, tomosynthesis‐Shimadzu metal artifact reduction technology (T-SMART) of the pelvis was performed at each visit. And the T-SMART images were evaluated by two senior surgeons (Y L and CQ T) independently to assess osseointegration. The functional outcomes were assessed according to the Musculoskeletal Tumor Society (MSTS) 93 score at the last follow-up.

## Results

The mean follow-up time was 35.3 months (range 28–45 months). There was no intraoperative complication. Negative surgical margins were achieved in all patients.

**Fig. 4:**
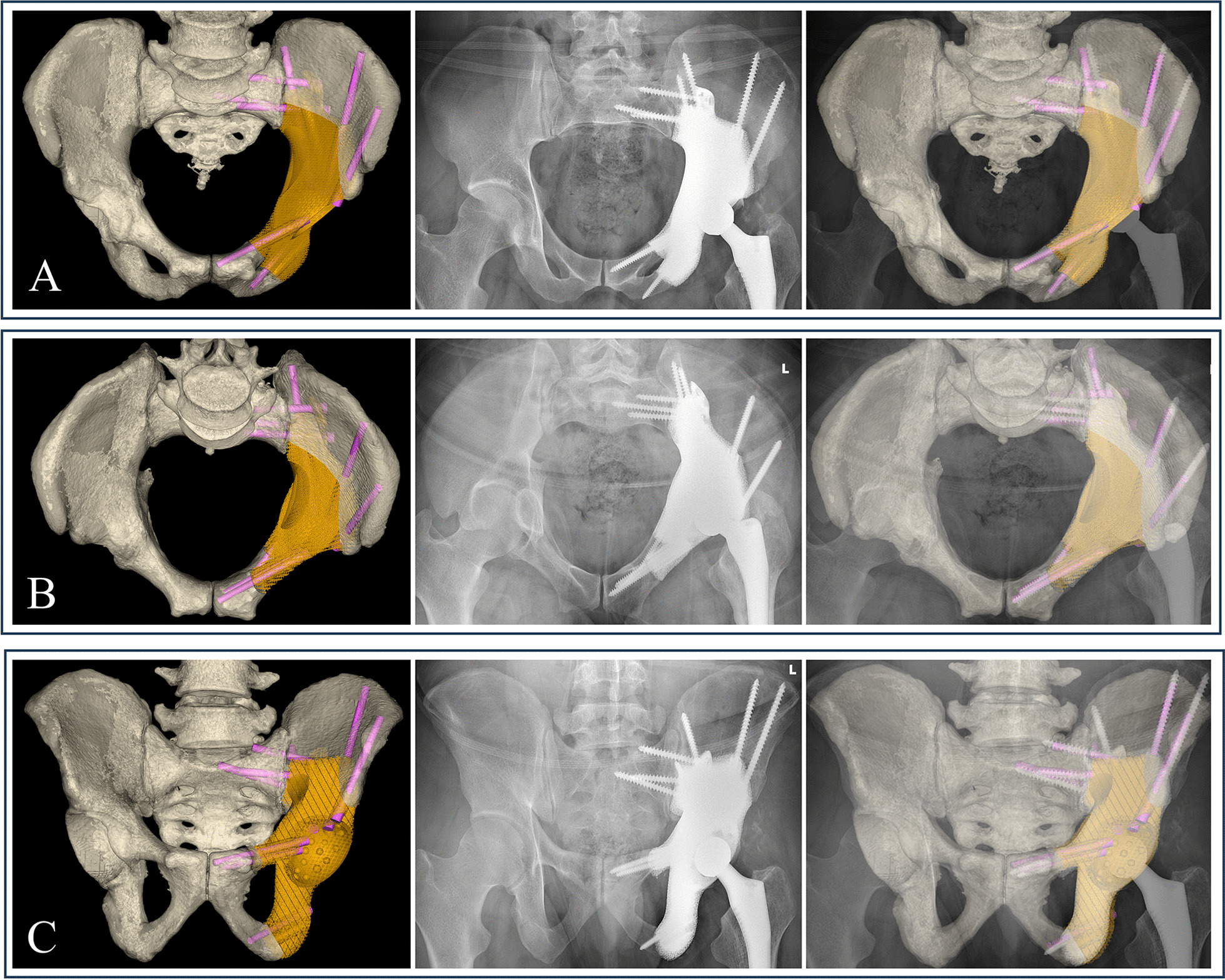
Accuracy evaluation. Postoperative anteroposterior **A** inlet, **B** and outlet **C** pelvic radiograph demonstrated precise reconstruction

**Fig. 5:**
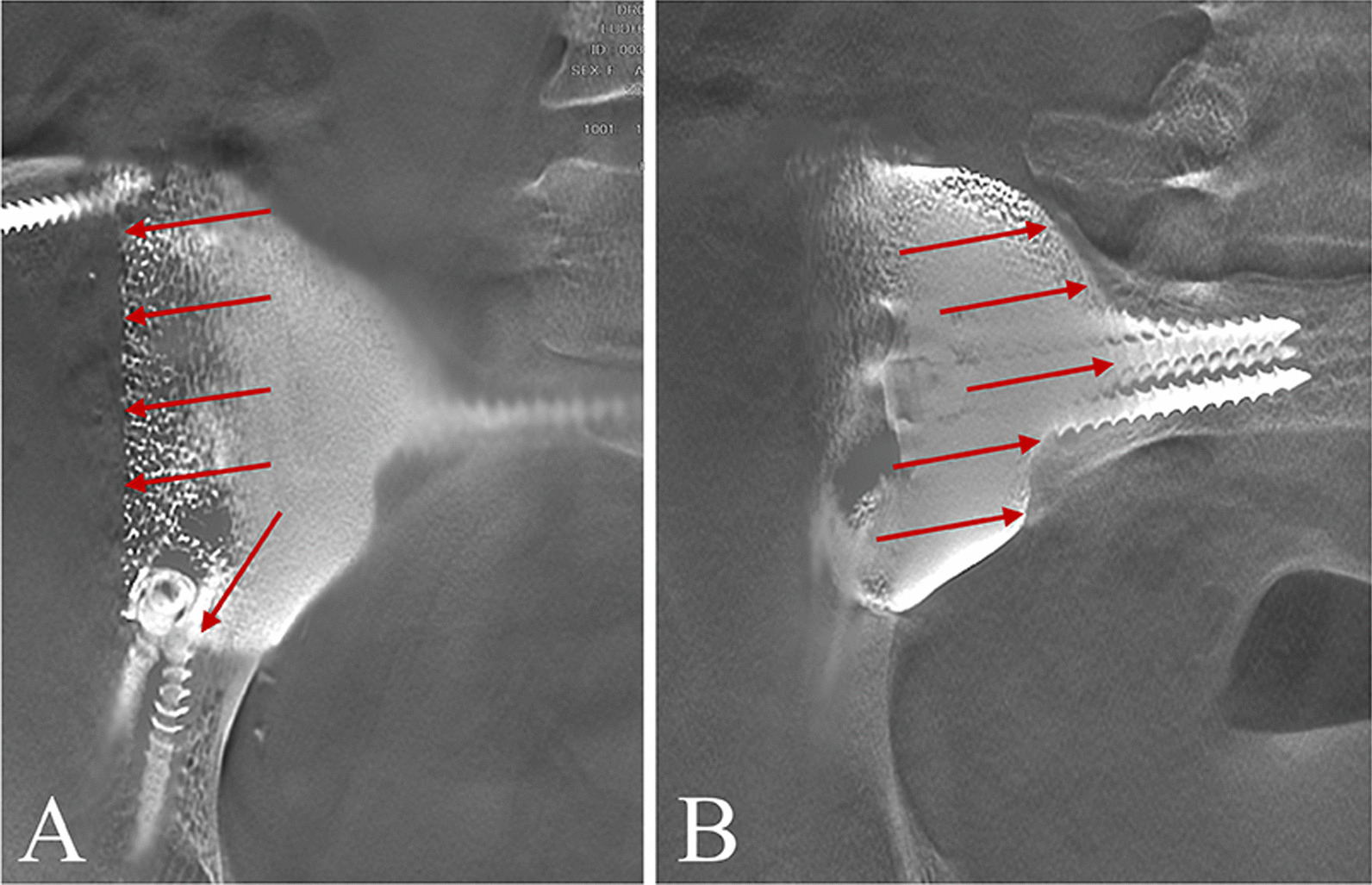
T-SMART images three months after the surgery showed the porous implant osseointegrated well with the residual ilium (**A**) and sacrum (**B**)

Postoperative pelvic radiographs showed that 3DPIs matched the shape and size of the bone defect. The anteroposterior, inlet, and outlet pelvic radiograph demonstrated precise reconstruction, consistent with the surgical planning (Fig. 4). In addition, T-SMART images showed good osseointegration at an average of three months after surgery (range 2–4 months) (Fig. 5).

There was no local recurrence or tumor metastasis. The average MSTS score was 24.4 (range 23–27). Delayed wound healing was observed in one patient, and the wounds healed after debridement. Prosthesis-related complications were not detected during the follow-up, such as aseptic loosening or structure failure.

## Discussion

In this study, seven patients with aggressive pelvic GCT were treated by wide resection assisted with PSBCGs and thereafter reconstructed by 3DPIs. Preliminary findings indicated that this technique was feasible and provided good clinical outcomes and reasonable function.

Advanced-stage pelvic GCTs are often accompanied by pathological fractures and destructed bones, and wide resection is usually recommended for these patients [23, 24]. In our cases, the lesions destructed the cortical bone with pelvic instability and discontinuity; therefore, wide resection was planned. It is worth noting that pelvic GCT is located deep and often difficult to detect early, and the tumor is often voluminous when seeking medical attention [24]. Combined with the complex anatomy of the pelvis, resection of such voluminous lesions is difficult when solely dependent on the surgeon’s experience and skill [25]. To overcome these problems and facilitate safe resection, PSBCGs were prepared for each patient preoperatively. Assisted with PSBCGs, wide resection with safe margins was achieved in all patients. The morphology of the excised specimen is consistent with the preoperative plan. And the pathological examination results of the cutting-edge status of the specimen were all negative. During the follow-up, no local recurrence was detected. All these results indicate that resection of lesions assisted with PSBCGs was a safe and feasible approach in the treatment of pelvic GCTs.

The key points of designing the PSBCGs were accurate planning and minimal error. Firstly, unlike malignant bone tumors in the pelvis with extensive surrounding edema area which requires resection of massive bone, the treatment of pelvic GCTs can preserve relatively more bone stock to decrease surgical morbidity. Therefore, the technique “multiplanar osteotomy with limited margins” was selected [14, 22]. The borders of PSBCP were designed consistent with these osteotomy plains. In addition, the internal surface of PSBCG perfectly fitting the cortical bone was designed to minimize error. Intraoperatively, the PSBCG could not slide on the bone cortex after moving to the target position.

Compared with patients with pelvic malignant tumors, patients with pelvic GCTs have a better prognosis and do not have the pressure of radiotherapy and chemotherapy. Therefore, patients have a higher demand for reconstruction in pursuit of good function and long-term reliable stability. However, subsequent pelvic reconstruction following wide resection is challenging due to the geometrical complexity of the pelvis. Until now, few publications have specifically addressed pelvic defects after wide resection of GCTs [7, 13, 14, 24, 26] (Table 2). The common choices for pelvic reconstruction included recycled tumor bone, modular pelvic prosthesis, and rod fixation. However, these traditional technologies were often accompanied by complications and poor function. 3DPIs used in our cases bear several outstanding advantages. Firstly, the implants were relative to the patient’s anatomy. The intraoperative photographs and postoperative radiological examinations showed perfect matching of 3DPIs with pelvic defects. Secondly, these anatomical 3DPIs could restore mechanical conduction based on the matched shape and size. In detail, 3DPIs after tumor resection in the area I reconstructed the mechanical conduction of the sacroiliac joint by connecting the sacrum to the residual ilium. And 3DPIs after tumor resection in area II (including II + III and I + II + III) reconstructed the mechanical conduction of the iliofemoral joint by the artificial hip replacement. Thirdly, the porous structure of the 3DPIs could assist in soft tissue attachment and insertion. This would reduce periprosthetic dead space, which is believed to help prevent the occurrence of infection [27]. In the present study, the use of 3DPIs with porous structure reduced the risk of infection compared with other reconstructive methods. In addition, 3DPIs with porous structure also could solve the integration between the implant and host bone, which is essential for the long-term stability of the prosthesis [28].

**Table 2:** Comparison of clinical and functional outcomes of various treatment studies

Study	Number of patients, n	Location, n	Methods of reconstruction, n	Follow-up, (months)	Local recurrence	Complications, n	Function, (MSTS score)
Current study	7	P I, (3); P II, (4)	3D-printed personalized implant	35.3	No	Delayed wound healing, (1)	24
Guo et al. [13]	14	P II, (14)	Recycled tumor bone, (3); Modular pelvic prosthesis, (11)	45	No	Delayed infection, (1) Bone nonunion, (1) Dislocation, (1) Wound healing disturbance, (4)	22
Zheng et al. [26]	21	P I, (8); P II, (7); P III, (6)	Without reconstruction, (13); Rod fixation and total hip arthroplasty; (5) Pelvic ring reconstruction; (3)	42	2/21	Delayed infection, (1) Dislocation, (1) Wound healing problem, (4)	22
Xiao et al. [14]	7	P II, (7)	Autogenous femoral head bone grafts, (7)	38.1	1/7	No	29
Verma et al. [7]	1	P I, (1)	3D-printed personalized implant	NA	NA	NA	NA
Khal et al. [24]	1	P II, (1)	3D-printed personalized implant	24	No	No	28

P I: lesion only involving pelvic I area; P II: lesion involving pelvic II area (including I + II, I + II + III, II + III); P III: lesion only involving pelvic III area; *NA* not available

Nowadays, 3DPI with porous structure is a very appealing choice for promoting osseointegration [29–31]. And T-SMART is often selected to evaluate the osseointegration of the bone–implant interface. This technique is known to provide good radiographic views of the bone–implant interface [32, 33]. In the present study, we also observed the trabecular structures connected to the implant surface through T-SMART to assess whether there was good osseointegration. To get high-quality T-SMART images, the patients were repositioned during radiography to ensure that the bone–implant interface was as perpendicular as possible to the examination platform. In the present study, to promote bone growth in the porous structure, the long axis of the screw aligned with the mechanical conduction axis and pressurized against the bone–implant interface. On the basis of relying on screws to achieve stability of the pelvic ring, interface compression helps with bone ingrowth. Postoperatively, T-SMART images showed good osseointegration in all patients.

Certain limitations of the present study should be noted. Firstly, this is a single-institution experience, with the operations carried out by one surgeon. In addition, this is a retrospective study with no control group or control group. Secondly, the number of patients is relatively small because pelvic GCTs are extremely rare. Thirdly, the follow-up is short, and complications might occur with a longer follow-up.

## Conclusions

The treatment of aggressive pelvic GCTs through wide resection assisted with PSBCGs and subsequent reconstruction with 3DPIs is a feasible method, which provides good clinical results and reasonable functional outcomes.

## Data Availability

The datasets used during the current study are available from the corresponding author upon reasonable request.
